# Assessing language abnormalities using NLP methods in speech excerpts of individuals at ultra-high risk for bipolar disorder

**DOI:** 10.1192/j.eurpsy.2025.647

**Published:** 2025-08-26

**Authors:** E. Kizilay, B. Arslan, E. Bora

**Affiliations:** 1 Department of Neurosciences; 2 Department of Psychiatry, Dokuz Eylul University, Izmir, Türkiye; 3 Department of Psychiatry, University of Melbourne and Melbourne Health, Melbourne. Australia

## Abstract

**Introduction:**

Detection for individuals at ultra-high risk for bipolar disorder (UHR-BD) is crucial due to the exploration of potential biomarkers at the early stages of bipolar disorder, including language abnormalities. Formal thought disorder (FTD) is an important symptom that can be observed in BD, which may be mildly noticeable during the early stages of the disease. Automated methods have demonstrated the ability to evaluate FTD in psychotic disorders and can also be employed to evaluate FTD in the speech of individuals at UHR-BD.

**Objectives:**

This study aimed to investigate the differences in language between UHR-BD and healthy controls (HC) using natural language processing (NLP) methods.

**Methods:**

We collected speech samples from 20 individuals at UHR-BD and 20 HC during descriptions of eight Thematic Apperception Test (TAT) pictures, which were then manually transcribed. After transcribing the text, word2vec was used to convert it into vectors. The semantic similarity between words was calculated using a moving window approach to windows of words sized 5-10. Finally, the mean and variance of similarities were determined.

**Results:**

The variances of similarities in the windows of 5 to 9 were increased in UHR-BD (p=0.004, p=0.005, p=0.01, p=0.02, and p=0.037, respectively). There was no significant difference regarding the mean similarity.

**Conclusions:**

To our knowledge, this is the first study to evaluate language with NLP methods in individuals at UHR-BD. Our findings showed that the variance of semantic similarity differed between the two groups. This indicates NLP methods may be used in the UHR-BD group to detect FTD.

**Disclosure of Interest:**

None Declared

